# Hospital admissions for dental disorders in patients with severe mental illness in Southeast London: A register‐based cohort study

**DOI:** 10.1111/eos.12752

**Published:** 2021-02-03

**Authors:** Jaya Chaturvedi, Wael Sabbah, Jennifer E. Gallagher, Jonathan Turner, Charlotte Curl, Robert Stewart

**Affiliations:** ^1^ King's College London London UK; ^2^ City University of London London UK; ^3^ King's College Hospital NHS Foundation Trust London UK; ^4^ South London and Maudsley NHS Foundation Trust London UK

**Keywords:** dental care, mental health, primary health care, psychotic disorders, risk factors

## Abstract

In people with mental disorders, adverse general health is well recognized but dental diseases remain underinvestigated. The objective of this study was to investigate risk factors for hospital admissions for dental disorders in patients with severe mental illness (SMI) and/or depressive disorder. De‐identified electronic mental health records from the South London and Maudsley NHS Foundation Trust (SLaM) were linked to national Hospital Episode Statistics (HES) data for analysis. Data were extracted for adults with a diagnosis of SMI (schizophrenia, schizoaffective disorder, bipolar disorder) and/or depression, who had received care at SLaM between 1 January 2010 and 31 March 2017. In the cohort of 18,999 patients thus obtained, the following factors were independently associated with hospital admission for dental disorders: female gender [odds ratio (OR) = 1.48, 95% CI: 1.31–1.68)], Health of the Nation Outcome Scales (HoNOS) problem drinking/drug taking (OR = 1.12, 95% CI: 1.05–1.19), HoNOS physical illness/disability (OR = 1.18, 95% CI: 1.12–.25), diabetes (OR = 1.24, 95% CI: 1.06–1.43), recorded current/past smoking (OR = 1.35, 95% CI: 1.06–1.43), treatment with antidepressant medication (OR = 1.48, 95% CI: 1.31–1.68), and depressive disorder (OR = 1.36, 95% CI: 1.11–1.68). Building on previous research in this population, which indicated a relatively high risk of acute care hospitalizations with dental disorders as discharge diagnoses, a number of demographic and clinical characteristics were found to be independent predictors over a 7‐yr period. Further research into these predictors would facilitate a better understanding of how adverse dental outcomes might be prevented.

## INTRODUCTION

Severe mental illness (SMI) is defined as a mental, behavioural, or emotional disorder that results in serious functional impairment which, in turn, interferes with (or limits) major life activities [1]. Both depression and SMI may result in erosion of functionality over time. With the English National Health Service (NHS), people with mental disorders, institutionalized or in the community, are entitled to the same degree of care as the general population but continue to have higher levels of morbidity and mortality [2, 3]. In the past decade, there has been a general improvement in oral health in high‐income countries [4], including a fall, from 46% in 1998 to 28% in 2009, in the prevalence of tooth decay among adults in the UK [5]; however, marked inequalities in oral health persist [6]. Higher rates of dental diseases have been described in people with mental disorders, who consequently have higher dental‐treatment needs [7]. Daily oral hygiene self‐care in this population may be impaired by reduced motivation or decreased access to dental services as a result of financial disadvantage [8], while some studies have also reported that mental illnesses and the medications used to treat them have a substantial and direct influence on dental diseases [9]. For example, many psychotropic medications are known to cause salivary gland hypofunction or xerostomia (dry mouth), an important risk factor for dental caries [10]. Smoking habits also contribute to the worsening of oral hygiene and the development of periodontal disorders [9] and there is a high prevalence of smoking in people with SMI [3].

Accordingly, we investigated dental status in a large historic cohort formed from a pre‐existing data linkage between mental health care and general hospital records, focusing on dental caries, tooth extraction, trauma, and oral mucosal lesions in patients with SMI and/or depression. A recent study, conducted using information from the same linked clinical databases that were used to provide data for the present study, reported that dental caries was a common cause of multiple hospital admissions among patients with SMI; an age‐ and gender‐standardized admission ratio of 2.55 was calculated for this event [11]. Preventing deterioration of oral health in patients receiving mental health care has been shown to lessen health‐care expenditure and to reduce the stress associated with the treatments required to manage underlying dental disorders [12]: these benefits would be consistent with aspirations articulated in the recently published NHS Long‐Term Plan [13] and have potential to reduce the financial burden of avoidable illness [14]. The aim of this study was to provide a clearer understanding of the associations between severe mental illnesses and dental disorders, thereby providing a basis for determining appropriate preventive oral health measures for use in mental health institutions.

## MATERIAL AND METHODS

The South London and Maudsley NHS Foundation Trust (SLaM) is one of the largest mental healthcare providers in Western Europe. It serves around 1.36 million residents across four boroughs (Lambeth, Southwark, Lewisham, and Croydon) in southeast London and provides a full range of secondary care services as well as some specialist national services. All clinical records in SLaM services have been electronic since 2006 (including legacy data from earlier years imported for some services) and have been made available for research since 2008 through SLaM's Clinical Record Interactive Search (CRIS) platform, which was set up and supported subsequently by the National Institute for Health Research Biomedical Research Centre at SLaM and King's College London. The CRIS platform has been described in detail [15] and essentially enables research access to full, but de‐identified, data from the electronic mental health record within a robust, patient‐led governance model, and with ethical approval for secondary analysis (Oxford C Research Ethics Committee, Reference 18/SC/0372). Over 150 peer‐reviewed research papers have been published to date using SLaM's CRIS data resource, including a number focusing on physical health outcomes in people with mental disorders [11, 16, 17, 18, 19].

This cohort study was designed to investigate the risk factors for hospital admissions with dental disorders in patients with SMI and/or depressive disorder. The data for this study were extracted via the CRIS platform. Data on hospital admissions were extracted from Hospital Episode Statistics (HES) which have been linked to CRIS data. Hospital Episode Statistics is a national data resource covering all NHS hospitals in England: it includes data on hospital admission episodes for both acute and mental health services and associated diagnoses recorded at discharge [20]. The cohort was defined as anyone who had received care at SLaM between 1 January 2007 and 31 December 2009, and who had received a diagnosis of SMI and/or depression recorded prior to 31 December 2009 and who was alive on 1 January 2010. Inclusion diagnoses comprised schizophrenia [International Statistical Classification of Diseases and Related Health Problems – 10th Revision (ICD‐10) F20x], schizoaffective disorder (ICD‐10 F25x), bipolar disorder (ICD‐10 F31x), and depressive disorder (depressive episode or recurrent depressive disorder; ICD‐10 F32x/ICD‐10 F33x), where x refers to any suffix to the code for inclusion of sub‐classes, such as F20.0 to F20.9. The follow‐up period for the cohort ran from 1 January 2010 to 31 March 2017. Only patients over 18 yr of age at the start (index) date were included.

### Measurements

Demographic information collected included age at index date, gender, and ethnicity. Neighbourhood‐level deprivation was assigned at the level of the Lower Layer Super Output Area 2011 (LSOA11) using the Index of Multiple Deprivation (IMD) 2010 score for that address unit [21]. The IMD is an official measure for the relative deprivation of small areas in England based on seven census‐derived attributes/indicators, ranking each LSOA from 1 (most deprived) to 32,844 (least deprived); LSOAs comprise approximately 1500 residents or 650 households [22].

From primary and secondary diagnosis information, binary variables were generated indicating the recording or not of schizophrenia (ICD10: F20.0–F20.9), schizoaffective disorder (ICD10: F25.0–F25.9), bipolar disorder (ICD10: F31.0–F31.9), and depressive/recurrent depressive disorder (ICD10: F32.0–F33.9). If a patient had more than one diagnosis, they were counted in each diagnosis group and were not mutually exclusive to each group. In addition, data obtained using recorded Health of the Nation Outcome Scales (HoNOS), an instrument routinely administered in UK mental health services, were extracted. The HoNOS scales were developed by the Royal College of Psychiatrists as a measure of health and social functioning [23]. For the analyses reported here, HoNOS subscales for alcohol/substance use and physical ill‐health were used.

Binary variables for psychotropic medication indicated whether patients were recorded as receiving any antipsychotic, hypnotic, or antidepressant medications within the follow‐up period of 1 January 2010 to 31 March 2017. This information was automatically extracted from free text using a bespoke natural language processing algorithm [15]. Data on the presence or absence of recorded smoking (current/past smoking combined) and presence or absence of recorded diabetes were also generated using natural language processing algorithms. Patients for whom data on smoking was missing (26.7%) were combined with the group labelled as having no recorded history of smoking because preliminary analyses indicated ‘missing completely at random’; that is, there was no association between smoking status being missing and any other variable in the dataset [24]. Information on obesity was not available in the records; however, body mass index (BMI) scores recorded closest to the index date were extracted and were categorized as follows: underweight (BMI <18.5), healthy weight (BMI 18.5–24.99), overweight (BMI 25–29.99), and obese (BMI ≥ 30) [25].

The primary outcome was hospital admission, for which dental events were recorded as a finding in patients admitted to hospital for any reason, regardless of whether the dental event was a primary or a secondary diagnosis. Dental event is defined as a combination of diagnosis and treatment variables associated with a recorded inpatient episode; for this definition, the relevant diagnoses were dental caries (ICD10: K02.0–K02.9), trauma (fractures, ICD10: S02.4–S02.9; wounds, ICD10: S01.4–S01.5; and dislocations, ICD10: S03.0–S03.5), or oral mucosal lesions, and relevant treatment was extraction (any procedures for extraction of teeth for any reason). The oral mucosal lesions variable was created based on categorization from a study on common oral mucosal diseases by Fedele *et al*. [26]. The dental lesions included in this category were denture stomatitis (ICD10 K12.1), denture hyperplasia (ICD10 K06.2), median rhomboid glossitis (ICD10 K14.2), candidiasis (ICD10 B37.0), herpetic gingivostomatitis (ICD10 B00.2), recurrent aphthous ulceration (ICD10 K12.0), and non‐specific ulcers (ICD10 K12.3) (1). Relevant diagnoses were sought from all the listed diagnosis and treatment variable fields.

### Statistical analyses

After describing the cohort, exposure–outcome associations were investigated. Descriptive statistical analyses and logistic regression models were conducted at patient level using STATA 13.0 [27]. Three logistic regression models were constructed to assess independence: Model 1 contained age, gender, ethnicity, IMD, HoNOS subscales for alcohol/substance use and physical ill‐health, smoking, and diabetes; Model 2 included the same covariates as Model 1 as well as three groups of medication – hypnotics, antidepressants, and antipsychotics; Model 3 contained the covariates from Model 2 and the mental disorder diagnoses.

## RESULTS

After applying the inclusion criteria, data on 18,999 patients were extracted from the CRIS database. The characteristics of the cohort and the percentages of the cohort with at least one dental event are presented in Table 1. Details of the dental outcomes are explained in the following paragraph. Within this cohort, the majority (53.5%) were female. The most common ever‐recorded diagnosis was of depressive/recurrent depressive disorder (54.6%). The mean ± SD age of the patients in the cohort was 46 ± 17 yr, and the highest proportion (39.9%) was in the 30‐ to 50‐yr age group. Almost two‐thirds (61.2%) of the cohort had recorded evidence of smoking history (current/past), and 16.9% had a recorded diagnosis of diabetes. Smoking history was missing for 26.7% of the cohort. Body mass index scores had not been recorded for more than half (58.1%) of the cohort, and so this variable was not included in any further analysis. Almost half (48.5%) of the patients in the cohort were recorded as receiving an antipsychotic agent.

**TABLE 1:** Detailed characteristics of the cohort of patients with severe mental illness (SMI) and depression (*N* = 18,999) and proportion of the cohort with at least one dental event

Characteristics	*N*	% with at least one dental event
Age group (yr)		
18–24	1480	10.5
25–34	3490	7.0
35–50	7597	7.1
51–60	2885	7.4
61–70	1550	6.6
>70	1997	6.8
Gender		
Male	8828	6.1
Female	10,171	8.4
Ethnicity		
British	8679	8.1
Irish	596	8.6
African/Caribbean	5287	7.5
Asian/South Asian	1075	5.2
Any other White background	1458	5.9
Other ethnicity	1276	5.3
Not stated	628	5.1
Index of Multiple Deprivation National Decile Score		
1	1049	7.1
2	5868	7.7
3	5061	7.3
4	2786	7.3
5	1711	6.1
6	767	8.6
7	449	8.5
8	440	6.8
9	296	3.4
10	132	7.6
Not stated	440	7.3
HoNOS subscale: Problem drinking/drug taking		
No problem	12,298	6.9
Minor problem requiring no action	1503	8.7
Mild problem but definitely present	1047	8.2
Moderately severe problem	746	10.5
Severe‐ to very‐severe problem	219	9.1
Not recorded	3186	7.2
HoNOS subscale: Physical illness or disability		
No problem	8778	6.4
Minor problem requiring no action	2829	7.9
Mild problem but definitely present	2317	8.4
Moderately severe problem	1500	9.9
Severe‐ to very‐severe problem	448	7.6
Not recorded	3127	7.2
Recorded smoking		
Yes	11,619	8.0
No	7380	6.2
Diabetes		
Yes	3205	8.9
No	15,794	7.0
Recorded medication		
Antidepressant	7138	9.4
Antipsychotic	9222	7.5
Hypnotic	5985	8.9
Previous diagnosis		
Schizophrenia	6739	6.2
Schizoaffective	1735	7.0
Bipolar	2720	7.3
Depressive	10,379	8.1

Of the full cohort of patients with SMI, 2.3% (*n* = 2670) had at least one hospitalization with a dental diagnosis and 1.9% (*n* = 2301) had undergone at least one dental operative procedure. These outcomes were overlapping: almost two‐thirds (65.1%) of patients with dental diagnoses also underwent dental operative procedures. Of the total hospital admissions for the cohort (*N* = 118,238), 1.1% had dental caries listed in the discharge diagnoses and 1.1% had extractions (operative procedure) listed; similarly to dental diagnosis/dental operative procedures, dental caries and extractions were not mutually exclusive. Among all admissions within the cohort with dental disorders listed in the discharge diagnoses (*n* = 3218), caries was recorded in 40.0% and trauma in 5.9%. Among admissions for whom dental operating procedures were recorded, extraction of tooth/teeth was evident in 64.6%. The remaining operating procedures recorded were: restorations (13.8%); other oral surgery procedures, such as excision of lesions and other unspecified operations (10.7%); periodontal procedures (5.8%); and other procedures relating to the tooth/mouth, such as diagnostic imaging and recording of jaw relationships (3.6%), fracture reductions (1.5%), and orthodontics (0.04%). The oral mucosal lesions variable included candidiasis (8.1% of all dental diagnosis), lesions of oral mucosa (3.1% of all dental diagnoses, including hyperplasia, non‐specific ulcers, and other unspecified lesions of oral mucosa), and other disorders of supporting structures (6.4% of all dental diagnoses, including median rhomboid glossitis, herpetic gingivostomatitis, recurrent aphthous ulceration, and denture stomatitis). As these were counts of admissions, it is possible that a single patient had multiple admissions.

Logistic regression models are summarized in Table 2. In unadjusted analyses, dental hospitalization was more common in younger patients, female patients, patients with higher scores on HoNOS subscales for problem drinking/drug taking and physical illness/disability, patients with diabetes, those receiving antidepressant treatment, and in patients with a diagnosis of depression. Hospitalization was less common in three of the minority ethnic groups than in those recorded as White British. In Model 1, associations persisted for age (negative), female gender, the three minority ethnic groups (negative), problem drinking/drug taking, physical illness/disability, and diabetes, and the association with smoking became stronger and statistically significant. In Model 2, all of these remained associated with the outcome with positive associations observed independently for antidepressant use and negative associations observed independently for antipsychotic use. In Model 3, replacement of medications with diagnoses did not alter associations with other covariates, and the association with depression persisted independently from the unadjusted model. In addition, another logistic regression model (Model 4) was run; in this Model, both a diagnosis of depression and antidepressant use were included with Model 1 covariates. This resulted in a largely unchanged odds ratio for depression diagnosis. When looking at a diagnosis of depression and antidepressant use independently of each other and of the remaining covariates (Model 4), use of antidepressant medication showed a very similar odds ratio to that of a diagnosis of depression.

**TABLE 2:** Logistic regression findings for variables predicting hospital admissions for dental events in patients with severe mental illness (SMI) (*N* = 18,999)

Variables	Logistic regression models				
	Unadjusted	Mutually adjusted			
		Model 1	Model 2	Model 3	Model 4
Age	0.96 (0.93–0.99)	0.90 (0.86–0.93)*	0.90 (0.86–0.94)*	0.90 (0.87–0.94)*	0.90 (0.87–0.94)*
Gender					
Male	1 (reference)	1 (reference)	1 (reference)	1 (reference)	1 (reference)
Female	1.40 (1.25–1.57)*	1.48 (1.31–1.68)*	1.40 (1.24–1.60)*	1.37 (1.20–1.56)*	1.37 (1.20–1.56)*
Ethnicity					
White British	1 (reference)	1 (reference)	1 (reference)	1 (reference)	1 (reference)
White Irish	1.06 (0.79–1.43)	0.96 (0.69–1.35)	0.95 (0.68–1.33)	0.95 (0.68–1.33)	0.95 (0.68–1.33)
African/Caribbean	0.92 (0.81–1.04)	0.84 (0.72–0.97)	0.92 (0.79–1.07)	0.95 (0.81–1.10)	0.95 (0.81–1.10)
Asian/South Asian	0.62 (0.47–0.82)	0.65 (0.48–0.88)	0.68 (0.50–0.92)	0.70 (0.51–0.94)	0.70 (0.51–0.94)
Any other White background	0.71 (0.56–0.90)	0.75 (0.59–0.97)	0.77 (0.60–0.99)	0.77 (0.60–0.98)	0.78 (0.60–1.01)
Other ethnicity	0.64 (0.49–0.83)	0.63 (0.47–0.84)	0.65 (0.49–0.87)	0.65 (0.48–0.86)	0.65 (0.48–0.87)
Index of Multiple Deprivation					
National Decile Score	0.98 (0.95–1.01)	0.99 (0.96–1.03)	0.99 (0.96–1.03)	0.99 (0.96–1.03)	0.99 (0.96–1.03)
HoNOS subscales (per unit increase)					
Problem drinking/Drug taking	1.13 (1.07–1.20)*	1.12 (1.05–1.19)*	1.11 (1.04–1.18)*	1.12 (1.05–0.19)*	1.12 (1.05–0.19)*
Physical illness or Disability	1.13 (1.08–1.19)*	1.18 (1.12–1.25)*	1.17 (1.10–1.23)*	1.16 (1.10–1.23)*	1.16 (1.09–1.22)*
Smoking	1.17 (0.98–1.39)	1.35 (1.06–1.43)*	1.33 (1.15–1.56)*	1.51 (1.31–1.76)*	1.51 (1.21–1.63)*
Diabetes	1.30 (1.14–1.49)*	1.24 (1.06–1.43)	1.23 (1.05–1.44)	1.34 (1.15–1.56)*	1.34 (1.09–1.49)
Recorded medication					
Antidepressant	1.62 (1.45–1.80)*	—	1.44 (1.26–1.64)*	—	1.34 (1.18–1.52)*
Antipsychotic	1.06 (0.95–1.18)	—	0.82 (0.70–0.95)	—	—
Hypnotic	1.38 (1.24–1.55)*	—	1.14 (0.99–1.32)	—	—
Previous diagnosis					
Schizophrenia	0.77 (0.68–0.86)	—	—	0.86 (0.69–1.06)	—
Schizoaffective	0.95 (0.79–1.16)	—	—	0.96 (0.77–1.18)	—
Bipolar	1.01 (0.86–1.17)	—	—	1.02 (0.83–1.25)	—
Depressive	1.31 (1.17–1.47)*	—	—	1.36 (1.11–1.68)	1.38 (1.20–1.59)*

Values are given as odds ratio (95% CI).

Model 1 contained the key variables [age, gender, ethnicity, Index of Multiple Deprivation (IMD), Health of the Nation Outcome Scales (HoNOS) subscales for alcohol/substance use and physical ill‐health, smoking, and diabetes]; Model 2, as for Model 1 plus three groups of medication (hypnotics, antidepressants, and antipsychotics); Model 3, as for Model 2 plus mental disorder diagnoses; and Model 4, as for Model 1 plus antidepressant use and depression diagnosis.

* Significant at *P* < 0.001.

## DISCUSSION

We examined the association between a number of characteristics of patients with SMI and/or depression and hospital admissions involving dental ‘events’ (i.e., a diagnosis and/or a treatment relating to oral health). The main findings were that an occurrence of at least one of these events was more common in younger patients, women, those with recorded current or past smoking status, those with diabetes, patients rated as having problem drinking/drug taking, patients receiving antidepressant treatment, or those diagnosed with depressive/recurrent depressive disorder.

Reflecting on this study's strengths and weaknesses, the main strength was the data source. As the CRIS model extracts data directly from the electronic health records of patients, valuable ‘real‐world’ information is available on routine mental health care in large cohorts [15]. A major challenge encountered when using data from electronic health records, especially data on mental health, is that most of the information is generally recorded in text in unstructured fields, rather than in structured fields. This challenge has been addressed in the CRIS system by utilizing natural language processing methods to extract such data [15]. A further strength of the data source was the linkage established between CRIS and HES data, providing valuable hospitalization information which was used in this study. One of the weaknesses of this study was its exploratory and descriptive nature. Comparison with a control cohort of patients without mental disorders would have substantially increased the value of our findings; however, because of the inability to access such data within the catchment area of southeast London from which the patient cohort was sourced, this comparison was not conducted but will be addressed in future follow‐up studies. The basis of this study was to conduct additional investigations on the findings of the study by Jayatilleke *et al*. [11] on standardized admission ratios for hospitalizations in people with the same diagnosis of SMI disorders as in this paper and compared with the general population. The study found dental caries to be among the top 10 reasons for hospital admissions [11] and was what motivated the authors of this paper to explore the recordings of dental events in these admissions. Another limitation of the present study was that the diagnoses for patients were not mutually exclusive. Patients had overlapping SMI diagnoses during the time period of interest. Separating these out could potentially provide clearer associations between the different variables. Along with history of smoking in these patients, another important factor is the use of recreational drugs, such as cannabis smoking. This factor was excluded from the present study because of insufficient information on the use of recreational drugs amongst this cohort but should be considered in future work, especially because of the increased prevalence of cannabis use among patients with SMI [28, 29]. Similarly, it would be interesting to see the effect of alcohol consumption on the oral health of patients of this cohort because patients with SMI have been found to have a higher likelihood of being alcohol dependent [28], and the risk of oral cancer increases significantly when tobacco and alcohol are combined. However, as information on alcohol use is dependent on the patient reports, such data are not completely reliable and also were not systematically recorded in the data used in this study.

In terms of this particular study, when interpreting the findings, it is important to bear in mind that, regardless of diagnosis, the study cohort will have had relatively severe mental disorders in order to have received specialist mental health care and are not representative of wider populations whose mental disorders remain undiagnosed or managed in primary care alone. Nonetheless, they provide information on this important group known to routine mental health care. Considering the outcomes of the study, these are derived from hospitalization data, which indicate not only the dental health but also the presentation pattern of patients. It might be that some patients were diagnosed with a dental issue but did not undergo treatment because it might have been more appropriate for them to wait until they left the hospital and returned to their general dental practitioner or as an outpatient for the procedure. Therefore, the results should be considered as reflecting only the tip of the iceberg regarding dental issues in patients with SMI and/or depression. However, it is still an important finding and warrants further study using links to outpatient and primary care data. In addition, there was limited information on some potential confounding factors, such as individual‐level socioeconomic status and patterns of health‐care utility, particularly the ability of patients to access routine primary dental care and in a timely manner. Residual confounding is therefore likely. In the case of the observed association between antidepressant medication and dental outcomes, confounding by indication needs to be considered [30], although it seems unlikely that perceived risk of dental outcomes would have had substantial influence on the type of medication prescribed for mental health.

The logistic regression models deal with a composite outcome, which explains odds of admission for dental events, as well as presentation behaviours. While women show increased odds of dental events, this may reflect a combination of dental diseases being more common in women, as well as the possibility of women being more likely than men to present for care. This is in line with the findings of a local study by Al‐Haboubi *et al*. [6], in which inequalities regarding the use of dental services were investigated among adults in inner southeast London: it was found that women were 14% more likely than men to visit the dentist over a period of 2 yr. The study by Teng *et al*. [29], previously discussed, concerning the utilization of dental care among patients with SMI, involved a similar sized SMI population sample of 19,609 patients (while there were 18,999 patients in our cohort) and also found a predominance of women in patients with SMI (56.1% women and 52.8% men), which is in line with the SMI population used in our study (53.5% women and 46.5% men), although the cohort in the present study also included patients with depression. Moreover, patients in the 18‐ to 24‐yr age group experienced more dental events (10.4%) than did the oldest population (>70 yr of age), in which dental events decreased to 6.8%. Similar patterns were found in the study by Al‐Haboubi *et al*. [6], in which patients over the age of 65 yr visited their local dentist less frequently than did younder patients. This could indicate that with the severity of SMI being higher in younger adults, they might be more likely to present for treatment, or that the conditions in older adults are not so complex as to require hospital admission. Additionally, the dental conditions of older adults might be more manageable in the community, or they might have lost more of their teeth, thereby not requiring as much dental care as younger adults, as per the National Adult Dental Health Survey (1, 31). A study by Fratto *et al*. [32] found that in addition to dry mouth, some antidepressants led to gingival bleeding and burning mouth syndrome and concluded that further studies were required to understand the effects of psychotropic drugs on oral health better. This corroborates our findings which showed that patients with depressive disorders, as well as patients on antidepressants, had greater odds of dental events than the remainder of the cohort. This could also be compounded by the heightened risk of self‐neglect in depressive disorders (). Further investigation of diet and toothbrushing practices (including use of fluoride toothpastes) might be warranted, as recommended by Public Health England [33], particularly in light of admissions relating to dental caries or their sequelae.

The admissions for dental disorders observed in this cohort represent complex, late‐stage, and expensive dental care in highly vulnerable populations. Over 60% of the population had had extraction of teeth recorded, and while extractions are not always complex and expensive, their use on patients with SMI might require special settings and additional time commitments, even for simpler dental procedures, as well as being charged an inpatient tariff rather than a primary dental care tariff. This warrants further research to understand the risk factors better, and to narrow down on the specific dental disorders afflicting patients with SMI and/or depression. This can lead to preventive measures being implemented at SLaM and other mental health institutions, possibly by adding routine dental screening to the protocol, and even making a dentist available at the SLaM outpatients department. With the NHS pushing for preventive care, this would be a good step forward in that direction. As highlighted in a paper by Gallagher & Fiske [34], who examined the need for Special Care Dentistry based on a review of published literature, suggestions were made on how services could be delivered in the future. For example, there may be a need to target regular dental check‐ups for patient groups at risk [35], so that preventive measures can be put into place to reduce the need for extractions. The study addressed the challenges faced in serving the needs of the vulnerable groups within the society, and the need for better access to health services across all societal groups [34]. The importance of dental health, especially in the population of patients with SMI and depression, cannot be emphasized enough. Good dental health can lead to overall improvement in the general physical health of these patients and the avoidance of undue stress associated with dental pain or even undergoing dental extractions in a hospital setting.

## CONFLICTS OF INTEREST

No conflicts of interest.

## AUTHOR CONTRIBUTION

RS and JG conceived the idea and supervised all aspects of the study. Analysis was carried out by JC with guidance from WS and JT, CC provided invaluable input on details around dental care provided to patients with SMI. JC took the lead in writing the manuscript. All authors provided critical feedback and helped shape the research, analysis and manuscript.

## References

[1] National Institute of Mental Health . Mental Health Information. The National Institute of Mental Health Information Resource Center; 2017. https://www.nimh.nih.gov/health/statistics/mental‐illness.shtml. Accessed December 12, 2018.

[2] Griffiths J , Jones V , Leeman I , Lewis D , Patel K , Wilson K , *et al*. Oral health care for people with mental health problems guidelines and recommendations report of BSDH working group B S D H UNLOCKING BARRIERS TO CARE. Br Soc Disabil Oral Health. 2000;5–9.

[3] Scott D , Happell B . The high prevalence of poor physical health and unhealthy lifestyle behaviours in individuals with severe mental illness. Issues Ment Health Nurs. 32(9):589–97. 21859410 10.3109/01612840.2011.569846

[4] Shen J , Wildman J , Steele J . Measuring and decomposing oral health inequalities in an UK population. Community Dent Oral Epidemiol. 41(6):481–9. 23992442 10.1111/cdoe.12071 PMC3812409

[5] Steele J , O’Sullivan I . Adult Dental Health Survey 2009: Executive Summary. Dental Health Survey; 2009. https://files.digital.nhs.uk/publicationimport/pub01xxx/pub01086/adul‐dent‐heal‐surv‐summ‐them‐exec‐2009‐rep2.pdf. Accessed December 17, 2018.

[6] Al‐Haboubi M , Klass C , Jones K , Bernabé E , Gallagher JE . Inequalities in the use of dental services among adults in inner South East London. Eur J Oral Sci. 2013;121(3pt1):176–81. 23659240 10.1111/eos.12043

[7] Khokhar MA , Khokhar WA , Clifton AV , Tosh GE . Oral health education (advice and training) for people with serious mental illness. Cochrane Database Syst Rev. 2016;17(1):9. 10.1002/14651858.CD008802.pub3 PMC6457656 27606629

[8] Friedlander AH , Marder SR . The psychopathology, medical management and dental implications of schizophrenia. J Am Dent Assoc. 2002;133(5):603–10. 12036166 10.14219/jada.archive.2002.0236

[9] Velasco‐Ortega E , Monsalve‐Guil L , Ortiz‐Garcia I , Jimenez‐Guerra A , Lopez‐Lopez J , Segura‐Egea JJ . Dental caries status of patients with schizophrenia in Seville, Spain: a case‐control study. BMC Res Notes. 2017;10(1):50. 28100262 10.1186/s13104-016-2368-9 PMC5241932

[10] Velasco‐Ortega E , Segura‐Egea J‐J , Córdoba‐Arenas S , Jiménez‐Guerra A , Monsalve‐Guil L , López‐López J . A comparison of the dental status and treatment needs of older adults with and without chronic mental illness in Sevilla, Spain. Med Oral Patol Oral Cir Bucal. 2013;18(1):e71–5. 23229258 10.4317/medoral.18332 PMC3548649

[11] Jayatilleke N , Hayes RD , Chang C‐K , Stewart R . Acute general hospital admissions with serious mental illness. Psychol Med. 2018;48(16):2676–83. 29486806 10.1017/S0033291718000284 PMC6236443

[12] Atkins CY , Thomas TK , Lenaker D , Day GM , Hennessy TW , Meltzer MI . Cost‐effectiveness of preventing dental caries and full mouth dental reconstructions among Alaska Native children in the Yukon‐Kuskokwim delta region of Alaska. J Public Health Dent. 2016;76(3):228–40. 26990678 10.1111/jphd.12141 PMC5010502

[13] NHS England . NHS Long Term Plan » Overview and summary. NHS England. 2019; https://www.longtermplan.nhs.uk/online‐version/overview‐and‐summary. Accessed March 5, 2019.

[14] Wanless D .Securing our future health: taking a long‐term view final report; 2002. https://www.yearofcare.co.uk/sites/default/files/images/Wanless.pdf. Accessed March 5, 2019

[15] Perera G , Broadbent M , Callard F , Chang C‐K , Downs J , Dutta R , *et al*. Cohort profile of the South London and Maudsley NHS Foundation Trust Biomedical Research Centre (SLaM BRC) Case Register: current status and recent enhancement of an Electronic Mental Health Record‐derived data resource. BMJ Open. 2016;6(3):e008721. 10.1136/bmjopen-2015-008721 PMC4785292 26932138

[16] Chang C‐K , Chen C‐Y , Broadbent M , Stewart R , O’Hara J . Hospital admissions for respiratory system diseases in adults with intellectual disabilities in Southeast London: a register‐based cohort study. BMJ Open. 2017;7(3):e014846. 10.1136/bmjopen-2016-014846 PMC5372120 28360254

[17] Chang C‐K , Hayes RD , Broadbent M , Fernandes AC , Lee W , Hotopf M , *et al*. All‐cause mortality among people with serious mental illness (SMI), substance use disorders, and depressive disorders in southeast London: a cohort study. BMC Psychiatry. 2010;10:77. 20920287 10.1186/1471-244X-10-77 PMC2958993

[18] Chang C‐K , Hayes RD , Perera G , Broadbent MTM , Fernandes AC , Lee WE , *et al*. Life expectancy at birth for people with serious mental illness and other major disorders from a secondary mental health care case register in London. PLoS One. 2011;6(5):e19590. 21611123 10.1371/journal.pone.0019590 PMC3097201

[19] Kisely S , Quek L‐H , Jo P , Lalloo R , Johnson NW , Lawrence D . Advanced dental disease in people with severe mental illness: systematic review and meta‐analysis. Br J Psychiatry. 2011;199:187–93. 21881097 10.1192/bjp.bp.110.081695

[20] NHS Digital .Hospital Episode Statistics (HES) [Internet]. NHS Digital; 2019. https://digital.nhs.uk/data‐and‐information/data‐tools‐and‐services/data‐services/hospital‐episode‐statistics. Accessed March 30, 2020

[21] Noble M , Wright G , Smith G , Dibben C . Measuring multiple deprivation at the small‐area level. Environ Plan. 2006;38(A):169–85.

[22] Department for Communities and Local Government . The English Indices of Deprivation 2015‐Frequently Asked Questions (FAQs); 2016. https://www.gov.uk/government/statistics/english‐indices‐of‐deprivation‐2015. Accessed November 9, 2018

[23] Mental Health Partnerships . Health of the Nation Outcome Scales (HoNOS). Mental Health Partnerships; 1995. https://mentalhealthpartnerships.com/resource/health‐of‐the‐nation‐outcome‐scales‐honos. Accessed December 3, 2018

[24] Grace‐Martin K .How to diagnose the missing data mechanism. The Analysis Factor; 2014. https://www.theanalysisfactor.com/missing‐data‐mechanism. Accessed November 11, 2018

[25] Diabetes.co.uk . BMI calculator ‐ body mass chart, BMI formula and history; Diabetes.co.uk.; 2018. https://www.diabetes.co.uk/bmi.html. Accessed December 3, 2018

[26] Fedele S , Sabbah W , Donos N , Porter S , D’Aiuto F . Common oral mucosal diseases, systemic inflammation, and cardiovascular diseases in a large cross‐sectional US survey. Am Heart J. 2011;161(2):344–50. 21315218 10.1016/j.ahj.2010.11.009

[27] StataCorp . Stata statistical software: release 13. College Station, TX: StataCorp LP; 2013.

[28] Menezes PR , Johnson S , Thornicroft G , Marshall J , Prosser D , Bebbington P , *et al*. Drug and alcohol problems among individuals with severe mental illnesses in South London. Br J Psychiatry. 1996;168(MAY):612–9. 8733801 10.1192/bjp.168.5.612

[29] Teng P‐R , Lin M‐J , Yeh L‐L . Utilization of dental care among patients with severe mental illness: a study of a National Health Insurance database. BMC Oral Health. 2016;16(1):87. 27585979 10.1186/s12903-016-0280-2 PMC5009687

[30] Boston University School of Public Health . Residual confounding, confounding by indication, & reverse causality. Confounding and effect measure modification; 2016. http://sphweb.bumc.bu.edu/otlt/MPH‐Modules/BS/BS704‐EP713_Confounding‐EM/BS704‐EP713_Confounding‐EM4.html. Accessed December 12, 2018.

[31] NHS Digital .Adult Dental Health Survey 2009 ‐ Summary report and thematic series. Adult Dental Health Survey; 2011. https://digital.nhs.uk/data‐and‐information/publications/statistical/adult‐dental‐health‐survey/adult‐dental‐health‐survey‐2009‐summary‐report‐and‐thematic‐series. Accessed December 5, 2019

[32] Fratto G , Manzon L . Use of psychotropic drugs and associated dental diseases. Int J Psychiatry Med. 2014;48(3):185–97. 25492713 10.2190/PM.48.3.d

[33] Public Health England, Department of Health, NHS, The British Association for the Study of Community Dentistry . Delivering better oral health: an evidence‐based toolkit for prevention; 2017. Vital 5(1), 13. 10.1038/vital731

[34] Gallagher JE , Fiske J . Special Care Dentistry: a professional challenge. Br Dent J. 2007;202(10):619–29. 17534326 10.1038/bdj.2007.426

[35] National Institute for Health and Care Excellence . Overview | Dental checks: intervals between oral health reviews | Guidance | NICE. NICE Guidel; 2004. https://www.nice.org.uk/guidance/cg19. Accessed November 16, 2019. 31869036

